# Will there be a warning for the next pandemic?

**DOI:** 10.1126/sciadv.aef2194

**Published:** 2026-09-16

**Authors:** Justin Lessler, C. Jessica E. Metcalf

**Affiliations:** ^1^ Department of Epidemiology, University of North Carolina, Chapel Hill, NC, USA.; ^2^ UNC Carolina Population Center, Chapel Hill, NC, USA.; ^3^ Department of Epidemiology, Johns Hopkins Bloomberg School of Public Health, Baltimore, MD, USA.; ^4^ Department of Ecology and Evolutionary Biology, Princeton University, Princeton, NJ, USA.

## Abstract

How to best allocate resources to combat the threat of pathogen emergence remains an important open question. Using archetypes that characterize the link from genotype to fitness in both zoonotic reservoirs and humans, we show that, across a range of plausible conditions, emergences of pathogens with pandemic potential in humans are unlikely to be preceded by detectable, failed, attempts. Yet, the number of “failed” emergence events contains information about the emergence potential of zoonotic pathogens, and should modify our beliefs about the underlying fitness landscape. Our work suggests that the most important, modifiable, risk factors for emergence may be phenomena that alter fitness landscapes, such as viral ecology in bridge species and human immunological landscapes.

## INTRODUCTION

Emerging pathogens pose one of the greatest threats to human health and livelihoods. In 2020, the global spread of SARS-CoV-2 had devastating impacts on mortality, health, and social well-being. COVID-19 is estimated to have killed more than 27 million people globally (*1*). Other pandemics of the modern era have been no less devastating. The 1918 “Great Influenza” killed as many as 100 million (roughly 10% of the global population at the time), and HIV has killed more than 33 million since its discovery in 1983 (*1*). This is to say nothing of the countless millions who have died from pandemics occurring before 1900. It is almost assured we have not seen our last pandemic.

Extensive resources have been deployed to meet the challenge of pathogens with pandemic potential, though the amounts are miniscule compared to the costs of a pandemics. These include efforts to detect pandemics before they happen, through characterizing viruses circulating in nonhuman reservoirs [e.g., the USAID PREDICT/DETECT program (*2*, *3*)] and surveillance and response to “spillovers” from animals to humans of potentially pandemic pathogens (*2*, *4*). Biosecurity efforts are another critical element of pandemic defense, including on farms (*5*), where high animal density and the presence of “bridge” species may allow pathogens to adapt to human hosts (*6*); wet markets, where humans have frequent exposure to a wide range of animal species (*7*); and laboratories, where potentially pandemic pathogens are studied (*8*). Resources have also been dedicated to ensure rapid response to an emergent pandemic, with a particular focus on developing and stockpiling vaccines and medical countermeasures to pathogens identified as being likely pandemic threats (*9*, *10*).

Many of the pathogens on the list of likely pandemic threats are there because they have a proven ability to infect humans but have only had limited, if any, transmission between human hosts (*11*, *12*). One reason these are considered to be of concern is that human infection and limited transmission might provide opportunity for adaptation toward efficient spread in humans (i.e., a reproductive number, *R*, greater than 1) (*11*). For example, much attention is paid to Middle Eastern Respiratory Syndrome (MERS-CoV, a coronavirus infection of camels) and Nipah virus (an infection of bats), both of which display self-limiting chains of transmission in humans. In general, more frequent self-limiting chains of transmission in humans is considered to be evidence for increased concern for pathogen emergence, but there is another way to interpret this evidence: Repeated spillovers that fail to cause pandemic spread (or substantial epidemics) are actually evidence that a virus is incapable of such spread without substantial evolutionary or ecological change. Here, we review evidence on pathogen emergence events to date and develop a quantitative framework to evaluate the risk of emergence.

## RESULTS

Most of the successful pandemic viruses of the genomic sequencing era were only detected after they had achieved their pandemic potential (Table 1). Upon detection, both H1N1pdm and SARS-CoV-2 had the full complement of traits required for supercritical human-to-human transmission (i.e., *R* was already greater than 1). Meanwhile, pathogens detected during self-limited chains of transmission have generally failed to manifest as a serious threat. Mpox is an exception: Stuttering chains of transmission following cessation of smallpox vaccination (*12*) were reported for years before suspected endemicity in Nigeria in 2017 (*13*). The same mpox clade (clade II) later spread globally, with a concentration in MSM (men who have sex with men) populations, leading the WHO to declare a public health emergency of international concern in 2022 (*14*). However, it appears likely that the global spread of mpox has been driven by reduced immunity due to discontinuation of smallpox vaccination (*15*) and introduction of the disease to high-risk networks, rather than genetic change in the virus (more on this later) (*16*).

**Table 1.** List of pandemic pathogens (left) and spillover viruses (right). Only mpox appears in both categories [we do not include Zika, as it was endemic to human populations in Africa before global spread (*36*)].

“Successful” pandemic viruses*	Spillover viruses
H1N1pdm	SARS-CoV-1†
SARS-CoV-2	MERS-CoV
**Mpox** ‡	Nipah
	Ebola
	Hendra virus
	**Mpox**
	Influenza (H5N1, H7N9, H9N2, etc.)

* HIV is not included because, though it was detected in the genomic era, it was already widespread and had emerged before this era (*37*).

† Technically pandemic but quickly died out, likely due to effective control of hospital transmission (*38*).

‡ Concentrated in specific subpopulations and long-term trajectory unclear (*14*).

### Emergence on archetypal fitness landscapes

Pandemic pathogens emerge from populations of viruses evolving on interrelated fitness landscapes in humans and zoonotic hosts (*17*). Mutations needed for successful transmission in humans may have varying impacts on transmission in primary zoonotic hosts and/or intermediate hosts (referred to as bridge species), and clusters of mutations may interact in complex ways. Taking the example of avian influenza, at minimum three phenotypic changes are required to enable this virus to efficiently transmit between humans: First, the viral polymerase must be altered to allow the virus to exploit human cell machinery; second, a change in hemagglutinin (HA) is required for the virus to bind strongly to human cell surfaces in the upper respiratory tract; and third, changes in the HA protein are required to prevent destruction during airborne transmission (*18*). These phenotypic changes require multiple genetic mutations, each of which may interfere with the others. Some, such as shifts in HA binding, are likely to have negative impacts in the primary zoonotic host (*18*).

**Fig. 1.**
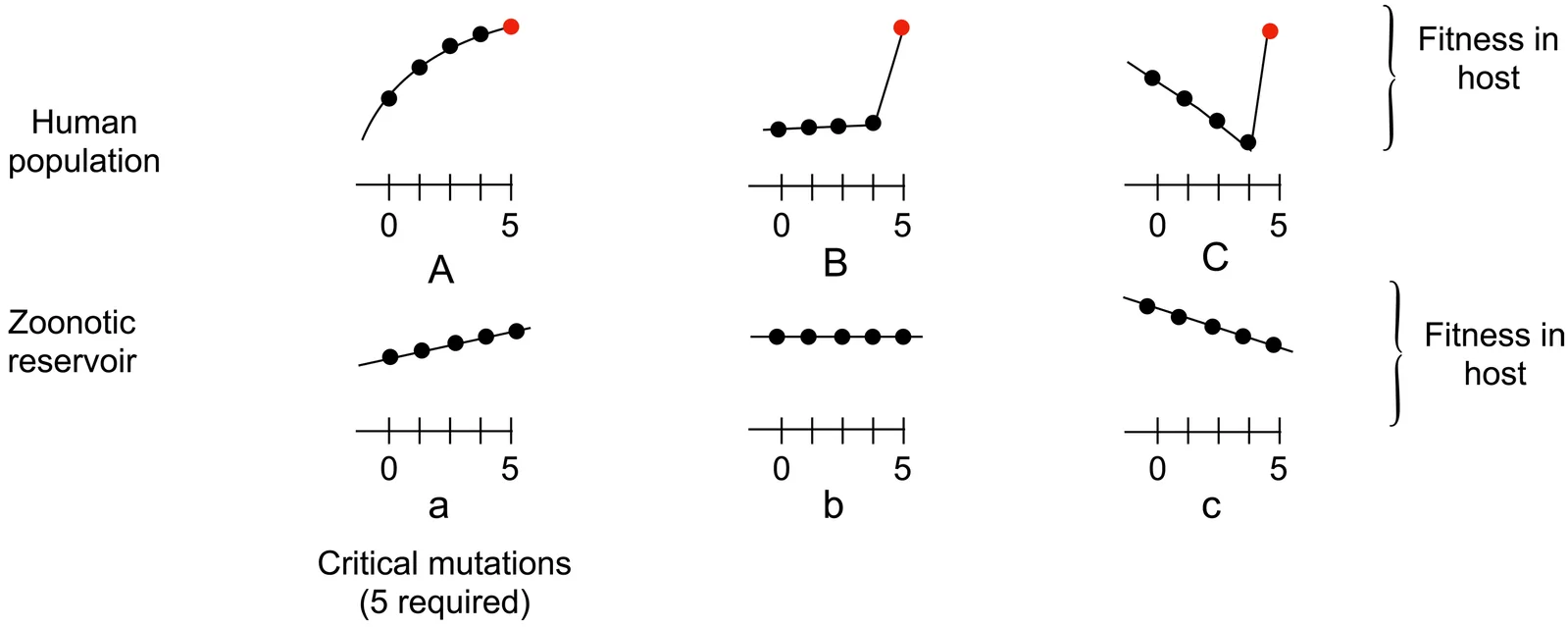
Fitness landscape archetypes. Fitness (*y* axis) as a function of the number of mutations that separate a virus from being an emergent and potentially pandemic risk pathogen with *R* > 1 in humans (*x* axis) assuming that five mutations are required. Fitness in the human host may be increasing gradually (A, “the hill”), increasing sharply (B, “the cliff”); and increasing sharply after a decline (C, “the valley”) until reaching criticality (depicted by the red dot). Fitness landscapes in the zoonotic reservoir may be (a) increasing, (b) flat (corresponding to neutral fitness), and (c) declining; a set chosen as reflecting the extremes of equilibrium distribution of mutations within zoonotic reservoirs.

To broadly characterize the emergence process, these complex landscapes can be reduced to a few archetypal patterns in human hosts (Fig. 1, A to C) and in the reservoir (Fig. 1, a to c). The mutations necessary for a virus to achieve criticality (represented by the red dot) might gradually increase viral fitness in humans as each mutation is acquired (a fitness hill, Fig. 1A), have a neutral fitness impact until the full complement of mutations is achieved (a fitness cliff, Fig. 1B), or even be individually detrimental until all are present (a fitness valley, Fig. 1C). Likewise, in the zoonotic host, each of these mutations could improve fitness (Fig. 1a), have a neutral impact on fitness (Fig. 1b), or decrease fitness (Fig. 1c). Of course, far more complex patterns are possible. In the human population, in particular, evolution might proceed in a variety of ways contingent on patterns of within and between host selection (*19*), and our framing effectively collapses many dimensions onto a single axis. Furthermore, although the magnitude and overall pattern of selection pressures is increasingly available [e.g., (*20*)], the nuance of the shape of fitness landscapes remains an important knowledge gap (*21*). Nevertheless, these archetypes can be used to help us understand the essentials of interacting landscapes between hosts.

**Fig. 2.**
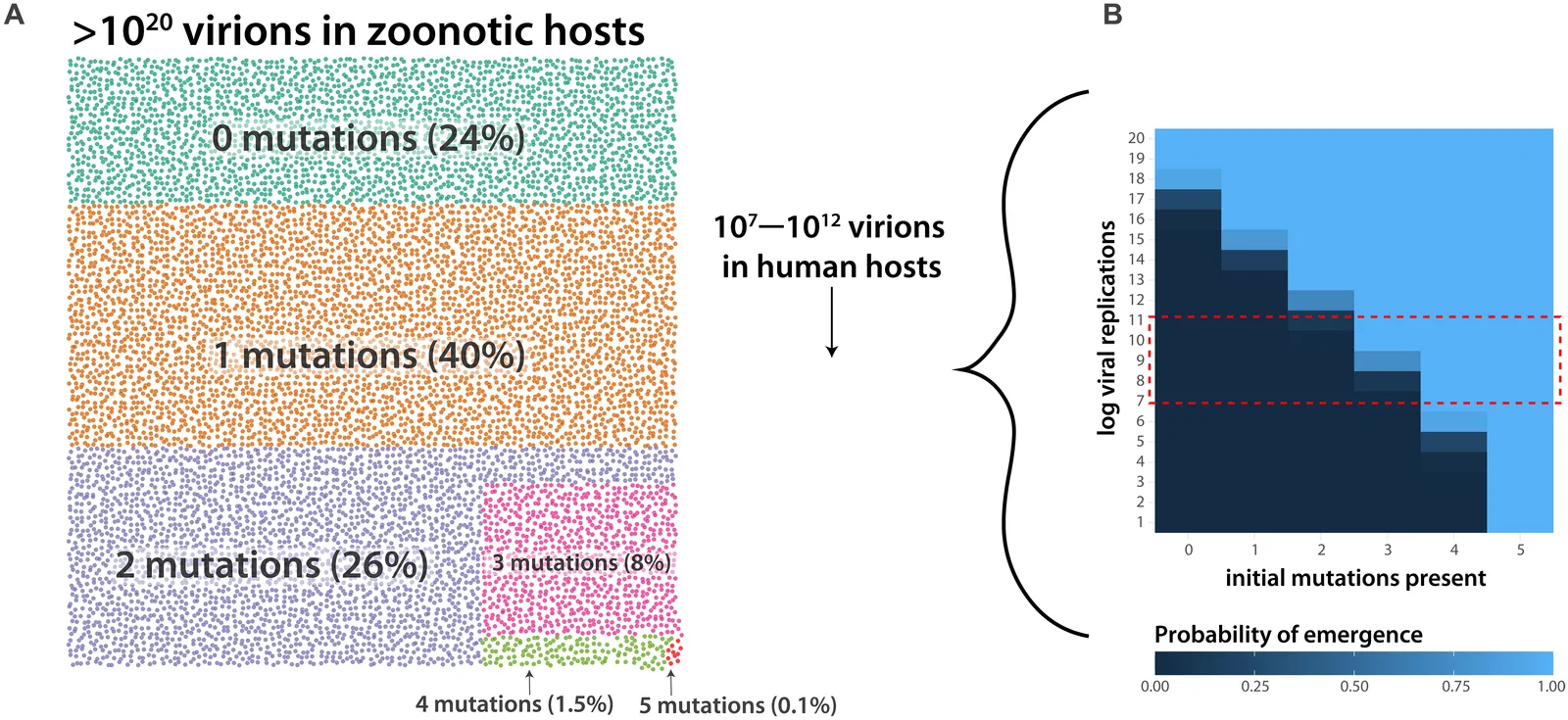
Probabilities of acquiring a set of five mutations needed for emergence under a neutral fitness landscape in the zoonotic host (Fig. 1a) and a fitness “cliff” (Fig. 1B) in humans. (**A**) The number of virions that occur in primary zoonotic hosts will likely be greater than 10^20^ (assuming on the order of 10^10^ infections with 10^10^ viral replications per infection), and approximately 10% of these virions will have at least three of the mutations needed for emergence in humans (order >10^19^ virions). By contrast, absent widespread circulation, we expect only 10^9^ to 10^12^ virions to be present in humans based on the viral load of individual infections. (**B**) The probability a virion that makes its way into humans from this animal population will result in a virus with pandemic potential varies with the number of mutations present upon entering the human host. Given a fitness cliff landscape, this probability is near 100% if four needed mutations are already present at typical viral loads for single infections; if three mutations are present, the probability will range from 0% to 100% depending on the viral load of the infection; and is near 0% across the range of reasonable viral loads if two or fewer mutations are present. The red dashed box indicates the range of the number of replication events that are likely to occur over the course of a human viral infection (10^9^ ± 2 logs) (*22*, *23*). See fig. S5 for a log scale version of (B).

The mathematics of viral evolution suggest that under many, if not most, of these patterns viruses will acquire the mutations needed for successful pandemic spread almost immediately upon entering a human host, or not at all. To simplify the problem, we consider a virus that needs five mutations to achieve its pandemic potential in humans. Under an assumption of a fitness cliff (Fig. 1B) where mutations have a neutral impact in humans until the full complement is acquired, the probability of achieving criticality after infecting a human host if the virus has four of the five needed mutations nears 100% at viral populations in the range seen in human infections (Fig. 2B, see Materials and Methods) (*22*, *23*). If the infecting virus has three of the five needed mutations, reaching criticality is less certain, but is almost assured at viral loads at the higher end of what is seen in human infections. However, if the virus has two or fewer of the necessary mutations, the probability of reaching criticality in a small number of human infections is almost zero. This relationship holds for both fitness hills and fitness valleys (patterns A and C), though the possibility of emergence is increased in the former and reduced in the latter (figs. S1 to S3).

Conversely, the sheer number of virions in zoonotic reservoirs versus human spillover hosts implies that potentially pandemic strains are far more likely to evolve in the zoonotic host unless selected against (tables S1 to S3). First, consider the example where the mutations needed to reach criticality in humans have a neutral fitness impact in zoonotic hosts (Fig. 1B). In this case, we expect 1.6% of the viruses in the zoonotic reservoir to be within at least one mutation of criticality (i.e., have four or more of the needed mutations, Fig. 2A), and almost 10% will have three or more of the required mutations. As a result, there is about a 10% chance under these conditions that a zoonotic infection would lead to near immediate emergence (figs. S1 to S5), an event roughly 450 times more likely than a human being infected with a virus with two or fewer required mutations and that virus evolving the required mutations (under the same conditions). This suggests that emergence might happen in several locations simultaneously, leading to a range of further challenges (*24*), although the evidence for this is so far minimal.

### A Bayesian approach to assessing emergence risk

**Fig. 3.**
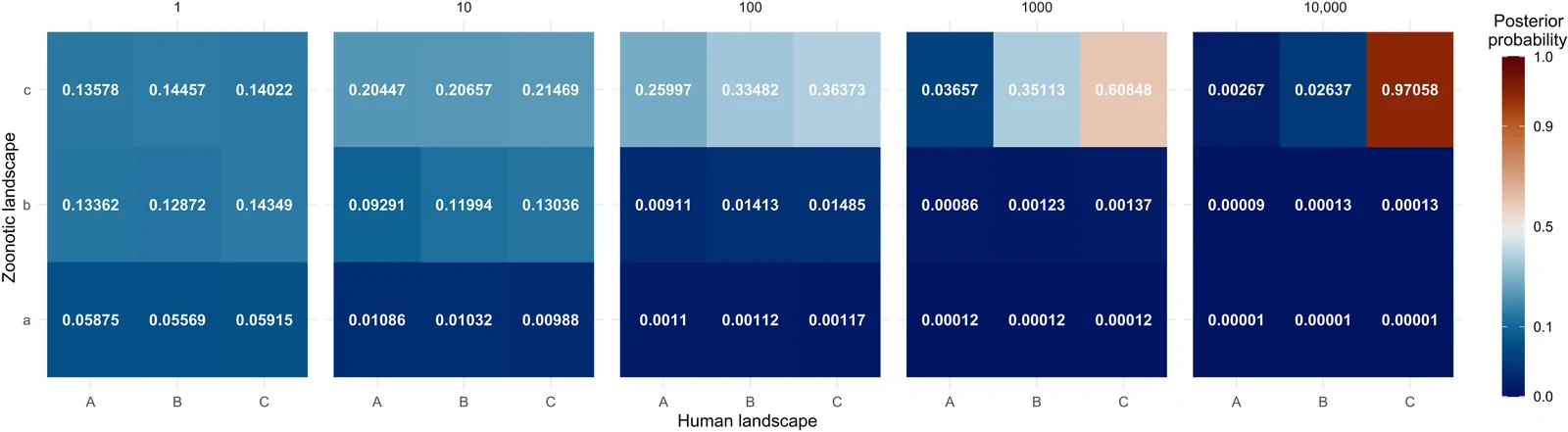
Bayesian updating to characterize fitness landscapes underlying emergence. Using landscapes identified in Fig. 1 as an exemplar (rows indicate zoonotic fitness landscapes, with a, indicating increasing fitness with each mutation, b, flat, and c, declining, and columns denote human fitness landscapes, with A, indicating a fitness hill, B, a fitness cliff, and C, a fitness valley), as the number of observed spillover events increases (panel headings), the posterior probability that an emergent pathogen maps to particularly underlying fitness landscapes can be defined (colors, numbers in boxes). Intuitively, because failed emergence is more likely when the virus is selected against, every failed emergence increases the probability of cells representing landscapes selecting against emergence (c and C) and decreases the probability of neutral and positive selection. After repeated failed emergence events, the probability of declining fitness in the reservoir and a fitness valley in human populations comes to dominate.

Considering these results in a Bayesian framework leads us to conclude that repeated self-limiting chains of transmission in humans should reduce our concern about particular viral lineages, not increase it (*25*). That is, each spillover event that does not lead to efficient human-to-human transmission of a virus reduces our estimate of the number of virions in the zoonotic reservoir that are on the edge of criticality. Rabies provides a concrete, if extreme, example of this logic: After millions of infections in humans over the centuries, no one worries about rabies’ efficient human-to-human spread. Thus, information from failed emergence events provides insight into the underlying fitness landscapes. In a simple example (see the Supplementary Materials for details), suppose that before seeing any failed emergence events, we consider all archetypical human and animal fitness landscapes to be equally likely (i.e., each combination has a probability of 0.1111). Seeing just one failed emergence event decreases our posterior probability of positive selection for pandemic mutations in the zoonotic host by almost half, but tells us little about the relative probability of other patterns (Fig. 3A). By the time we see 10 failed emergence events, our posterior probability of positive selection in the zoonotic host falls to around 1%, and we begin to believe that negative selection in the zoonotic host is nearly twice as likely as neutral selection (Fig. 3B), but little information is gleaned about possible human fitness landscapes. Once we reach 100 failed attempts, our posterior beliefs are such that positive and neutral selection in the zoonotic host are viewed as exceedingly unlikely (Fig. 3C) and we begin to discount the possibility of positive or neutral selection for pandemic mutations in human hosts. At 1000 failed emergences, we are quite confident that mutations are negatively selected for in zoonotics hosts, and that there is no positive selection in humans (Fig. 3D), and by 10,000 failed emergences, we have near certainty of negative selection in zoonotic hosts and a fitness valley in humans (Fig. 3E). This relationship holds regardless of the number of critical mutations required for human circulation, with neutral mutation being more rapidly discounted if we assume fewer necessary mutations and requiring more failed emergence to dismiss if we assume more (see figs. S6 and S7). Similar logic can be used to think about viral prevalences in the zoonotic species (see figs. S8 and S9). Such approaches, alongside research into the underlying mechanisms of host-virus interactions [e.g., contrasting SARS-CoV-2 and MERS (*21*)] have the potential to inform us as to this crucial knowledge gap.

In recent work, Simony and Kennedy (*25*) reached a similar conclusion through a different analytic approach, concluding that “novel” pathogens pose a greater risk than ones that frequently spill over into human hosts. Simony and Kennedy do not attempt to explicitly deal with evolutionary fitness landscapes and how our views of these (and zoonotic host prevalence) are shaped by failed emergences, but the changing posterior probabilities of evolutionary landscapes (and zoonotic prevalences) we calculate provide one mechanistic explanation for their results.

## DISCUSSION

Our analysis shows why prior spillover events that fail to spread widely may be the exception, rather than the rule, when it comes to pathogen emergence. We further show why increasing numbers of observations of such failed spillover events should lower, rather than increase, our concern that a pathogen lineage will reach pandemic potential. These results should lead us to focus on those factors that might allow pathogens to circumvent the probabilistic barriers to their emergence.

### Ecology and emergence risk

In our main analysis, we focused on evolutionary adaptation of viral lineages and ignored the role of ecology. Yet, ecological changes can also create the conditions necessary for supercritical transmission. A low prevalence pathogen of limited concern can become a high burden pathogen due to changes in contact patterns. For example, respiratory adenovirus infections are largely considered a negligible problem but were a chronic problem in military recruits prevaccination due to the close and sustained contact that occurs in army barracks (*26*). Similarly, the international spread of mpox in 2022 was associated with entry into high contact sexual networks (*14*). The spread of infectious disease in humans in general is likely associated with the emergence of cities and high-density populations (*27*). Such ecological changes might be a more frequent, more detectable, and easier-to-modify driver of emergence than viral evolution.

**Fig. 4.**
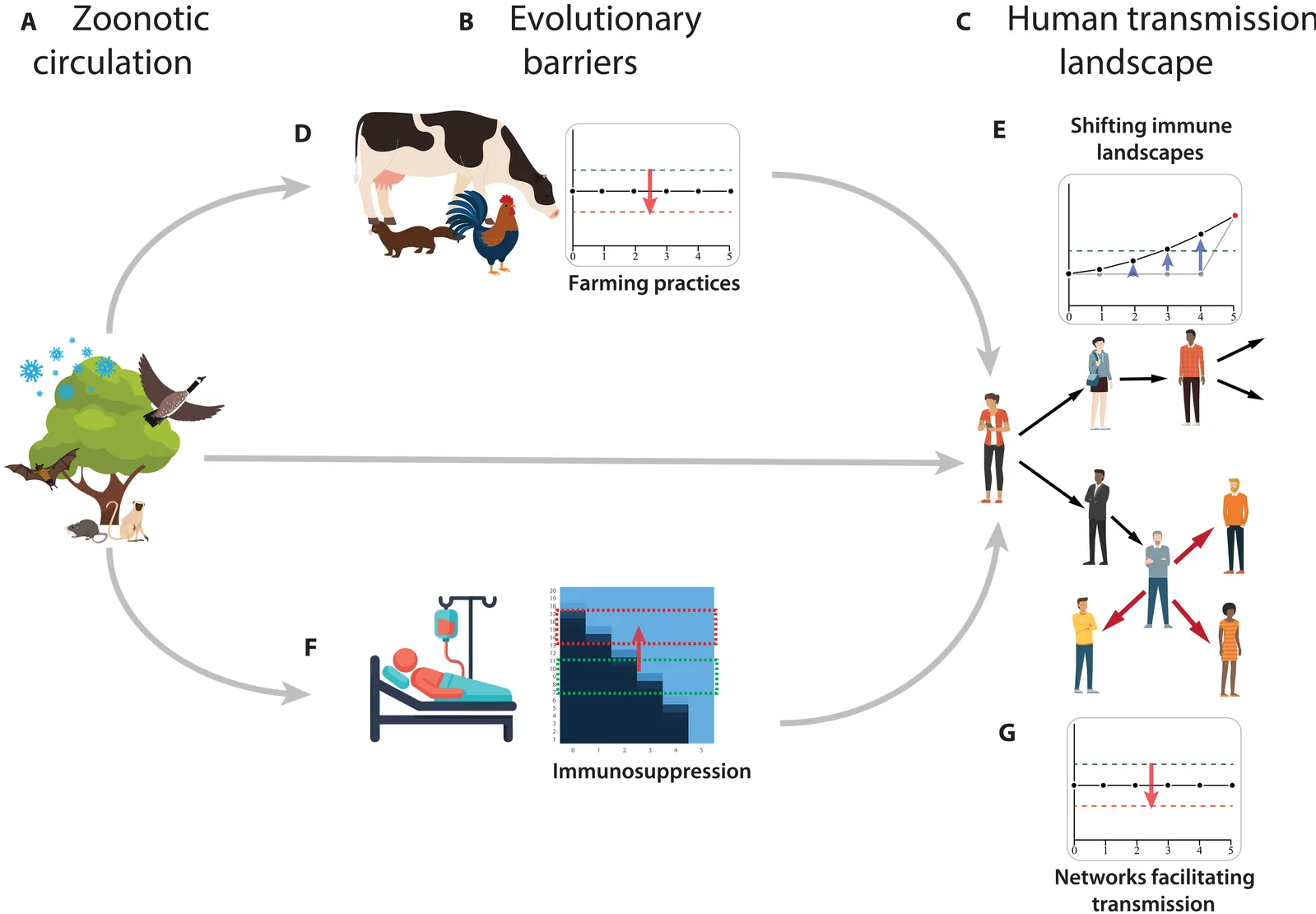
Ecological changes and pathogen emergence. Changing ecology can allow a virus with predominately zoonotic circulation (**A**) to overcome formerly (effectively) insurmountable barriers to emergence and cause pandemic spread. These include ecological changes that allow the virus to overcome evolutionary barriers (**B**) such as high density (and other) farming practices (**D**) that allow for circulation of the zoonotic virus in animal species where it would not be able to circulate absent these practices, potentially opening an evolutionary bridge to pandemic emergence. Immunosuppressed humans (**F**) may also serve as a pathway for a virus to reach its pandemic potential, by allowing for far more replications in a human host than would be seen in immunocompetent individuals, allowing the virus to overcome probabilistic barriers to emergence. The transmission landscape in humans (**C**) can also change due to changes in human ecology, such as shifting immunological landscapes (**E**) (e.g., the loss of smallpox immunity in humans) that allow viral mutants that would not be fit to effectively circulate in human hosts; and viral entry into (perhaps emergent) transmission networks (**G**) where it can effectively spread despite lacking the fitness to do so in the general population.

Farming practices, habitat change, and other factors that modify animal ecology can open new evolutionary pathways, creating potential bridges from zoonotic hosts to humans (Fig. 4). The recent H5N1 crisis in the United States is a prime example. Mammalian spillovers of highly pathogenic avian influenza have been detected for years, but never before has sustained transmission in a mammalian host been documented (*18*). The current epidemic in dairy cattle is likely possible only because of modern milking and farming practices (*28*) and may provide an opportunity for the virus to find evolutionary pathways bridging fitness valleys previously preventing emergence in humans (*29*). Similarly, H5N1 outbreaks have occurred in high-density mink farms and resulted in mutations thought to increase pandemic potential in humans (*30*). While most of these ecological changes will not result in a pandemic emergence, they may substantially increase the odds, and reset our expectations about the probability of emergence derived from past self-limiting spillover events.

This suggests that the clearest path to strengthening biosecurity against potentially pandemic pathogens is better characterizing modifiable drivers of viral ecology in zoonotic hosts, humans, and possible intermediate species. These drivers go beyond farming practices. For example, there is evidence that the interaction between land-use changes and a shifting climate lead to higher-density fruit bat populations, alongside increased viral loads due to physiological stress, potentially increasing ease of spread of Hendra virus in these populations (human-bat contact is also increasing, though we would argue that is less important) (*31*). This may change the zoonotic landscape of Hendra evolution and could potentially be mitigated through ecological countermeasures including habitat maintenance (*32*). Similarly, immunosuppression in humans is associated with chronic viral infection and may allow for viruses to reach criticality through increased (cumulative) viral population size and reducing the negative impact of some mutations (*33**,* *34*). Treatment of immunosuppression (e.g., antiretroviral therapy for HIV) reduces these opportunities (Fig. 4).

Changes may not be purely ecological. In particular, altered cross-species interactions may also open new evolutionary pathways, such as infection of hosts where recombination and reassortment are possible. For example, for influenza, the sialic acid receptor in pigs is accessible to both human and avian influenza strains (*35*), making pigs an important host where reassortment could occur. Such recombinations and reassortments essentially result in “teleportation” across fitness valleys, allowing a virus to acquire multiple mutations in one fell swoop, thereby circumventing some of the mathematics presented above. This, among other reasons (e.g., the ability to survive outside hosts), is why our analysis is less relevant to bacteria, where transfer of mobile elements is far more common, allowing quick acquisition of critical characteristics, such as drug resistance.

### Limitations and final thoughts

This work has several limitations. We focus on simplified, archetypal, fitness landscapes that do not reflect the rich landscape of pathogen evolution and may miss some situations where emergence may be more likely. However, even in those cases, the same Bayesian logic we apply suggests that we should down weight the probability that “easy” paths to emergence exist for a lineage for which we have observed failed emergences. Our analysis also collapses both within and between host processes into a single fitness landscape, and thus, we are not fully exploring the nuances how different selective pressures interact. Further, as noted, we ignore mechanisms that may allow pathogens to “teleport” across fitness valleys, and thereby more easily emerge; although here, too, beliefs about emergence risk to associate with numbers of “failed” emergence events (self-limiting spillovers) should be informed by the same Bayesian logic. Last, we only consider one form of evidence about emergence probabilities, self-limiting spillovers. Laboratory experiments and other lines of evidence may provide important information about underlying evolutionary landscapes and could be incorporated into similar probabilistic frameworks.

This work provides a more nuanced appreciation of how to interpret evidence from failed emergence events and a path to reframe how we think about self-limiting chains of transmission. It is clear that failed emergence events should be investigated and curtailed, because, as illustrated by COVID-19, the costs of pandemics are huge, and any efforts that even fractionally decrease their risk are likely cost-effective. However, particularly as failed emergence events accumulate, they should not induce panic or distract from broader approaches to pandemic prevention. Likewise, any evidence that a new ecological pathway to emergence may have opened, such as sustained circulation in a new species, should raise concern—even if the new pathway has yet to cause detected infections in humans. This is particularly the case as that first infection in humans may be as likely to cause a pandemic itself as it is to be the warning of a coming storm.

## MATERIALS AND METHODS

Expanded methods with embedded code are available in the Supplementary Materials.

### Calculation of prevalence in zoonotic species

To estimate the prevalence of viruses within zoonotic populations with a specific number of the critical mutations needed for efficient spread in the human population, we construct a fitness graph for each virus with the array of possible signatures of critical mutations (increasing, flat, and declining). We then run the system of difference equations corresponding to this graph to equilibrium, thereby obtaining the estimated background prevalence of each of the mutational signatures in the animal reservoir.

In more detail,

1. We construct a vector of viral fitnesses, **w** of length 2^*N*^, where *N* is the number of critical mutations that must be present for spread in the human population (for illustration, we set *N* = 5). Each of the entries in this vector corresponds to a bit signature where 1 indicates the presence of a particular critical mutation and 0 indicates absence. Hence, indexing from 0, each number in the array corresponds to a bit signature (e.g., for 5, in binary 00101, corresponds to the presence of the first and third required mutations), and the array entry corresponds to the relative fitness of a virus with that signature (see functions in PrevalenceCalculatingFunctions.R).

2. We then construct a matrix of transition probabilities Δ to and from states with a particular mutation probability. Under a very simple model of molecular evolution, where a single base mutation is needed at each site, this gives entries in Δ

$$\delta_{\mathrm{ij}}={\left(1-\frac{3}{4}m\right)}^{x_{11}}\times {\left(1-\frac{1}{4}m\right)}^{x_{00}}\times {\left(\frac{1}{4}m\right)}^{x_{01}}\times {\left(\frac{3}{4}m\right)}^{x_{10}}$$

where $\delta_{\mathrm{ij}}$ is the rate of movement between sequence state i and sequnce state j, m is the per-site mutation rate, $x_{11}$ denotes the number of critical mutations present in both i and j, $x_{00}$ is the number absent in both i and j, $x_{01}$ is the number absent from i and present in j, and $x_{10}$ is the number present in i and absent in j.

3. We then construct a system of difference equations such that

$$y_{t+1}=\frac{y_{t}\Delta w}{\sum \left(y_{t}\Delta w\right)}$$

and run it until equilibrium, defined as $\text{max}\left(|y_{i,t+1}-y_{i,t}|<\epsilon \right)$.

Below, we apply this to each of the zoonotic evolutionary patterns of fitness (increasing, flat, and declining), assuming that selection pressure is weak when present.

### Bayesian updating based on failed emergences

Within a Bayesian framework, we take the probabilities calculated above to help us understand how observed viral introductions into humans that fail to lead to emergence should change our beliefs about the likely evolutionary conditions and underlying prevalence of various mutation patterns in the zoonotic species.

### Posterior probability of fitness landscapes.

We want to calculate the probability of different fitness landscape combinations given a number of failed emergences. Using the probabilities calculated above for emergence under various fitness patterns and our assumptions about the distribution of viral population sizes in humans, we can use Bayesian inference to understand what underlying fitness landscapes are most likely.

We treat each pair of fitness landscapes (e.g., human fitness landscape A, zoonotic fitness landscape b) as a hypothesis, with equal probability of being true before the observation of a failed emergence. Using standard Bayesian approaches, in the rstan package, we obtain a posterior distribution of probabilities based on the chances of seeing x failed emergence attempts under each.

Specifically, we assume that the observed number of failures X follows a binomial distribution

$$X∼\text{BINOM}\left(1-\rho_{i},X\right)$$

where $\rho_{i}$ is the probability of successful emergence under fitness landscape hypothesis i (e.g., zoonotic landscape a and human landscape B). Emergence probablities, $\rho_{i}$, are calculated through simulation as detailed in the analysis above and under the assumption that the number of viral replications in a human host follows a normal distribution on a log 10 scale with a mean of 9 and a standard deviation of 2. Functionally, we assume we have a set of beliefs about the probability of each hypothesis being true, $p_{i}$, with a prior distribution of

p∼Dirichlet(α)

where $\alpha_{i}=1/9$. We then use standard MCMC techniques to estimate the posterior distribution of $p_{i}$ with the simplified overall observation probability (given a belief) of

$$X∼\text{BINOM}\left(1-\sum_{i}\rho_{i}p_{i},X\right)$$

It should be noted that while we illustrate our results using a relatively simple set of canonical hypotheses, this same approach could be used for any arbitrary number of hypotheses with arbitrary complexity.

### Posterior probability of zoonotic prevalence

We can use the same approach under assumptions about the human landscape of fitness of emergence in human populations (here, assuming pattern B) to update our beliefs about the patterns of zoonotic prevalence under a prior assumption that prevalence follows the patterns suggested by neutral mutation (pattern b). We use the same basic approach as above, except that $\rho_{i}$ now represents the probability of emergence in human fitness regime B given we start with i necessary mutations present, and $p_{i}$ now represents the assumed prevalence of virions with i mutations in the zoonotic host population.

## Supplementary Materials

### This PDF file includes:

Supplementary Materials and Methods Algorithms S1 to S13 Tables S1 to S4 Figs. S1 to S9
