# Supplementary material for: Will there be a warning for the next pandemic?
Source: Sci Adv. 2026 Sep 16;12(38):eaef2194. doi: 10.1126/sciadv.aef2194 (PMC13580539; doi:10.1126/sciadv.aef2194)
Supplement: Supplementary file 1 [file sciadv.aef2194_sm.pdf]

Science Advances
AAAS

Supplementary Materials for

Will there be a warning for the next pandemic?

Justin Lessler and C. Jessica E. Metcalf

Corresponding author: Justin Lessler, jlessler@unc.edu

Sci. Adv. **12**, eaef2194 (2026)
DOI: 10.1126/sciadv.aef2194

This PDF file includes:

Supplementary Materials and Methods
Algorithms S1 to S13
Tables S1 to S4
Figs. S1 to S9

```r
## Setup. Source utility functions and load packages.
library(tidyverse)
source("../code/PrevelanceCalculatingFunctions.R")
N_SIMS <- 50000000 #Actually run on cluster reduce to run locally
```

Supplementary Materials and Methods

1. Calculation of prevalence in zoonotic species

In order to estimate the prevalence of viruses with a certain number of the critical mutations needed for efficient spread in the human population, we construct a fitness graph [CITE] for virus with each possible signature of critical mutations. We then run the system of difference equations corresponding to this graph to equilibrium, thereby obtaining the estimated background prevalence of each of the mutational signatures in the animal reservoir.

In more detail we: 1. Construct a vector of viral fitnesses, $\mathbf{w}$ of length 2^N, where N is the number of critical mutations that must be present for spread in the human population (in this case N=5). Each of the entries in this vector corresponds to a bit signature where 1 indicates the presence of a particular critical mutation and 0 indicates absence. Hence, indexing from 0, each number in the array corresponds to a bit signature (e.g., 5 = 00101 or the presence of the 3rd and first mutations), and the array entry corresponds to the relative fitness of a virus with that signature. We then construct a matrix of transition probabilities Δ to and from states with a particular mutation probability. Under a very simple model of molecular evolution, this gives entries in Δ:

$$\delta_{ij} = \left(1 - \frac{3}{4}m\right)^{x_{11}} \times \left(1 - \frac{1}{4}m\right)^{x_{00}} \times \left(\frac{1}{4}m\right)^{x_{01}} \times \left(\frac{3}{4}m\right)^{x_{10}}$$

where δ_{ij} is the rate of movement between sequence state i and sequnce state j, m is the per site mutation rate, x_{11} denotes the number of critical mutations present in both i and j, x_{00} the number absent in both i and j, x_{01} the number absent from i and present in j n, and x_{10} is the number present in i and absent in j 3. We then construct a system of difference equations such that:

$$\mathbf{y}_{t+1} = \frac{\mathbf{y}_t \Delta \mathbf{w}}{\sum(\mathbf{y}_t \Delta \mathbf{w})}$$

and run it until equilibrium, defined as $\max(|y_{i,t+1} - y_{i,t}| < \epsilon)$.

Below, we apply this to each of the zoonotic evolutionary patterns, with very weak selective pressure when present.

Pattern a: Positive Selection in Zoonotic Host

First we calculate the zoonotic prevlances where each of the five necessary mutations increases fitness in the zoonotic host by 0.1%

Algorithm S1: Calculation of zoonotic prevalence under positive selection

```r
library(gt)

##First create the fitness vector for the five postive mutation fitness graph
.
fit_vect <- mutfit_to_graphfit(1.001^(0:5))

##Now calculate equilbrium prevalences
zoo_a_prevs <- calc_equilibrium_prev(5, fit_vect)

##convert back to a by mutation prevalence. `
zoo_a_prevs_critmuts <- graphprev_to_critprev(zoo_a_prevs)

zoo_a_prevs_critmuts_tbl <- zoo_a_prevs_critmuts %>%
    rename(`critical mutations`=crit_mut, `prevalence`=p) %>%
    gt() %>%
    fmt_scientific()

gt:::as.tags.gt_tbl(zoo_a_prevs_critmuts_tbl)
```

Table S1. Equilibrium prevalence (zoonotic host) of critical mutations under weak positive selective pressure in the zoonotic host (pattern a).

critical mutations	Prevalence
0.00	2.08×10^{-6}
1.00	1.32×10^{-4}
2.00	3.35×10^{-3}
3.00	4.25×10^{-2}
4.00	2.70×10^{-1}
5.00	6.84×10^{-1}

Pattern b: Neutral Mutation

We now consider the prevalence for a virus where there are five necessary mutations for a virus to achieve its pandemic potential in humans that have no impact on zoonotic fitness.

Algorithm S2: Calculation of zoonotic prevalence under neutral mutation.

```
##First create the fitness vector for the five neutral mutation fitness graph
.
fit_vect <- mutfit_to_graphfit(rep(1,6))

##Now calculate equilbrium prevalences
zoo_b_prevs <- calc_equilibrium_prev(5, fit_vect)

##convert back to a by mutation prevalence.
zoo_b_prevs_critmuts <- graphprev_to_critprev(zoo_b_prevs)



zoo_b_prevs_critmuts_tbl <- zoo_b_prevs_critmuts %>%
    rename(`critical mutations`=crit_mut, `prevalence`=p) %>%
    gt() %>%
    fmt_scientific()

gt:::as.tags.gt_tbl(zoo_b_prevs_critmuts_tbl)
```

Table S2. Equilibrium prevalence (zoonotic host) of critical mutations under neutral fitness landscape the zoonotic host (pattern b).

critical mutations	prevalence
0.00	2.37×10^{-1}
1.00	3.96×10^{-1}
2.00	2.64×10^{-1}
3.00	8.79×10^{-2}
4.00	1.46×10^{-2}
5.00	9.77×10^{-4}

Pattern c: Negative Selection in Zoonotic Host

Here we calculate the zoonotic prevlances where each of the five necessary mutations decrease fitness in the zoonotic host by 0.1%

Algorithm S3: Calculation of zoonotic prevalence under negative selection.

```
##First create the fitness vector for the five negative mutation fitness grap
h.
fit_vect <- mutfit_to_graphfit(.999^(0:5))

##Now calculate equilbrium prevalences
zoo_c_prevs <- calc_equilibrium_prev(5, fit_vect)
```

```
##convert back to a by mutation prevalence.
zoo_c_prevs_critmuts <- graphprev_to_critprev(zoo_c_prevs)

zoo_c_prevs_critmuts_tbl <- zoo_c_prevs_critmuts %>%
    rename(`critical mutations`=crit_mut, `prevalence`=p) %>%
    gt() %>%
    fmt_scientific()

gt:::as.tags.gt_tbl(zoo_c_prevs_critmuts_tbl)
```

Table S3. Equilibrium prevalence (zoonotic host) of critical mutations under weak negative selective pressure in the zoonotic host (pattern c).

critical mutations	prevalence
0.00	8.89×10^{-1}
1.00	1.06×10^{-1}
2.00	5.01×10^{-3}
3.00	1.19×10^{-4}
4.00	1.41×10^{-6}
5.00	6.72×10^{-9}

2. Calculate emergence probabilities in humans

In order to calculate the emergence probability in humans we perform stochastic simulations from a set of difference equations where the zoonotic infection is seeded at random from the zoonotic population and then evolves in humans for the designated number of viral replications. Viral replication is assumed to occur with a designated baseline reproductive number, R_0, placed under increasing immunological pressure such that the reproductive number R decays at some rate γ (i.e., $R_t = R_0(1-\gamma)^t$, where t is the current generation) calibrated such that the expected final size is N. Specifically:

1. γ is determined using an optimizer comparing the expected final size to some desired final size, N [`final_viral_pop`, `calc_decay_rate`]. The final viral populaition (equal to the total number of replications). The final population size is determined by calculation the generation, g, where the viral population falls below ϵ as:

$$g = \left\lceil \frac{-\left(\log R_0 - \log\left((1-\gamma)/2\right)\right) - \sqrt{\left(\log R_0 - \log\left((1-\gamma)/2\right)\right)^2 + 2\log(1-\gamma)\log\epsilon}}{\log(1-\gamma)} \right\rceil$$

based on which we can calculate the total viral popualation (i.e., total number of replications in humans + 1) given γ, $size(\gamma)$, as:

$$size(\gamma) = 1 + \sum_{i=1}^{g} R_0^i \,(1-\gamma)^{i(i-1)/2}$$

2. We now stochastically simulate evolution in the human host with transition probabilities and fitness vectors calculated as above [`sim_emergence_gen_decay`], with the following modifications:

    - The starting prevalence vector is based on a single introduction from the zoonotic host with probability proportional to the underlying zoonotic prevalence.
    - The next generation is determined by a Possion draw where the vector of rates corresponding to sequence states is calculated as:
    $$\boldsymbol{\lambda_{t+1}} = R_0(1-\gamma)^{t+1} N_t y_{t+1}$$
    where N is the total population size at the previous generation and y_{t+1} is the normalized prevalences for each state as calculated above.
    - The simulation runs until either (a) the virus becomes extinct or (b) a virus with all necessary pandemic mutations emerges.

We run this for all combinations of human and zoonotic mutation patterns and then examine the patterns of emergence. In the below simulation we calculate the emergence probability by different combinations of fitness landscapes, also tracking emergence by number of mutations initially present and other factors. We then examine these patters for each combination of human and zoonotic mutation.

The exact patterns of human fitness we consider are:

- A. Fitness Hill: A pattern where fitness increases by 10% for every mutation acquired on the pathway to achieving pandemic potential.
- B. Fittness Cliff: A pattern where fitness is constant until all mutations necessary to achieve pandemic potential are present.
- C. Fitness Valley: A pattern where fitness decreases by about 10% for every mutation acquired until all necessary mutations are present.

Algorithm S4: Calculation of emergence probabilities under different combinations of human and zoonotic fitness landscapes as well as number of viral replications.

```
### Lower N if this is to be run.

source("../code/PrevelanceCalculatingFunctions.R")
##Create a list of human and animal fitness landscapes.
zoonotic_fit_landscapes<- list( a = mutfit_to_graphfit(1.001^(0:5)),
                                b= mutfit_to_graphfit(rep(1,6)),
                                c = mutfit_to_graphfit(.999^(0:5)))
```

```
human_fit_landscape <- list(A = mutfit_to_graphfit(1.1^(0:5)),
                            B = mutfit_to_graphfit(rep(1,6)),
                            C =  mutfit_to_graphfit(c(0.9^(0:4),1)))



##Create zoonotic prevalences from fitness landscapes
zoonotic_prevs <- zoonotic_fit_landscapes ##create similar list where we will
overwrite contents
for (i in 1:length(zoonotic_fit_landscapes)) {
    zoonotic_prevs[[i]] <-  calc_equilibrium_prev(5, zoonotic_fit_landscapes[
[i]])
}

##Now create a 3-dimensional array of emergence probabilities [zoonotic, huma
n, log10 total viral load]
cell_R0 <- 10 #roughly middle of range for estimates.
sz_rng <- 1:20
decay_rates <- sapply(10^sz_rng, calc_decay_rate, R0=cell_R0)

# tmp <- sim_emergence_gen_decay(5, zoonotic_prevs[[1]], cell_R0, decay_rates
[10], max_gens=30, fit_vect=human_fit_landscape[[1]], n_sims=10000)
# print(graphprev_to_critprev(zoonotic_prevs[[1]]))
# print(mean(tmp[,"emerge_gen"]<Inf, na.rm=T))




emerge_df <- NULL
emerge_df_initmuts <- NULL



for (i in 1:length(zoonotic_prevs)) {
    for (j in 1:length(human_fit_landscape)) {
        for (k in 1:length(decay_rates)) {
            tmp <- sim_emergence_gen_decay(5, zoonotic_prevs[[i]], cell_R0, d
ecay_rates[[k]], max_gens=40, fit_vect=human_fit_landscape[[j]], n_sims=N_SIM
S)
            res <- tmp %>% as_tibble() %>%
                    summarize(p_emerge = mean(emerge_gen<Inf, na.rm=T),
                              cell_R0 = cell_R0,
                              zoo_landscape = names(zoonotic_prevs)[i],
                              human_landscape = names(human_fit_landscape)[j]
,
                              pop_sz_log10 = sz_rng[k],
                              decay_rate = decay_rates[k],
                              med_gen = median(emerge_gen)) %>% ungroup()
            emerge_df <- bind_rows(emerge_df, res)
```

```
            res_initmuts <- tmp %>% as_tibble()%>% group_by(init_muts) %>%
                    summarize(p_emerge = mean(emerge_gen<Inf, na.rm=T),
                              cell_R0 = cell_R0,
                              zoo_landscape = names(zoonotic_prevs)[i],
                              human_landscape = names(human_fit_landscape)[j]
,
                              pop_sz_log10 = sz_rng[k],
                              decay_rate = decay_rates[k],
                              med_gen = median(emerge_gen)) %>% ungroup()

            emerge_df_initmuts <- bind_rows(emerge_df_initmuts, res_initmuts)
        }
    }
}




## Loads the stored data.

emerge_df <- readRDS("emerge_df50M.rds")
emerge_df_initmuts <- readRDS("emerge_df_initmuts50M.rds")
```

To translate these results to an overall probability of mutation, we assume that the distribution of viral replications in humans follows a normal distribution on the log 10 scale with a mean size of 10^9 and a standard deviation of ± 2 logs.

Algorithm S5: Collapsing of emergence probabilities based on the assumed distribution of viral replications.

```
## Collapse the fitness based emergence probabilities based on a distribution
of final sizes
mn_log10_final_sz <- 9
final_sz_sd <- 2

emerge_probs <- emerge_df %>%
    group_by(zoo_landscape, human_landscape) %>%
    summarize(p_emerge = weighted.mean(p_emerge, dnorm(pop_sz_log10, mn_log10
_final_sz, final_sz_sd))) %>%
    ungroup()
```

Which gives us the following emergence probabilities for each landscape combination.

Algorithm S6: Plotting of fitness landscapes.

```
library(gt)

sum_tbl <- emerge_probs %>% mutate(probability = round(p_emerge, 4)) %>%
    mutate(zoo_landscape = paste("zoonotic landscape",zoo_landscape)) %>%
    select(-p_emerge) %>% rename(`human landscape`=human_landscape, `zoonotic
landscape`=zoo_landscape) %>%
    gt(groupname_col="zoonotic landscape") %>%
    tab_header(
        title="Probability of Emergence by Landscape"
    )

gt:::as.tags.gt_tbl(sum_tbl)
```

Table S4. Probability of emergence under different human and zoonotic landscapes given normally distributed viral total viral replications in humans on a log 10 scale with a mean of 9 and a standard deviation of 2.

Probability of Emergence by Landscape

human landscape	probability
zoonotic landscape a	
A	0.9705
B	0.9674
C	0.9684
zoonotic landscape b	
A	0.1251
B	0.0926
C	0.0797
zoonotic landscape c	
A	0.0049
B	0.0010
C	0.0005

We now can examine how the probability of emergence varies by the number of mutations present in the initially infecting virus for each human landscape. Note that the probability of emergence in humans should be independent of the zoonotic landscape given this number of mutations.

Algorithm S7: Comparison of emergence probabilities by initial mutations present.

```
emerge_probs_initmuts <- emerge_df_initmuts %>%
    group_by(human_landscape, init_muts) %>%
    summarize(p_emerge = weighted.mean(p_emerge, dnorm(pop_sz_log10, mn_log10
```

```
_final_sz, final_sz_sd))) %>%
    ungroup()


options(repr.plot.width = 8, repr.plot.height=6)
emerge_probs_initmuts %>%
    ggplot(aes(x=init_muts, y=p_emerge, color=human_landscape)) +
        geom_point() +
        geom_line() +
        ylab("Probability of Emergence") +
        xlab("Mutations Initially Present") +
        scale_color_discrete(name="Human Landscape") +
        theme_bw()


`summarise()` has grouped output by 'human_landscape'. You can override using
the `.groups` argument.
```

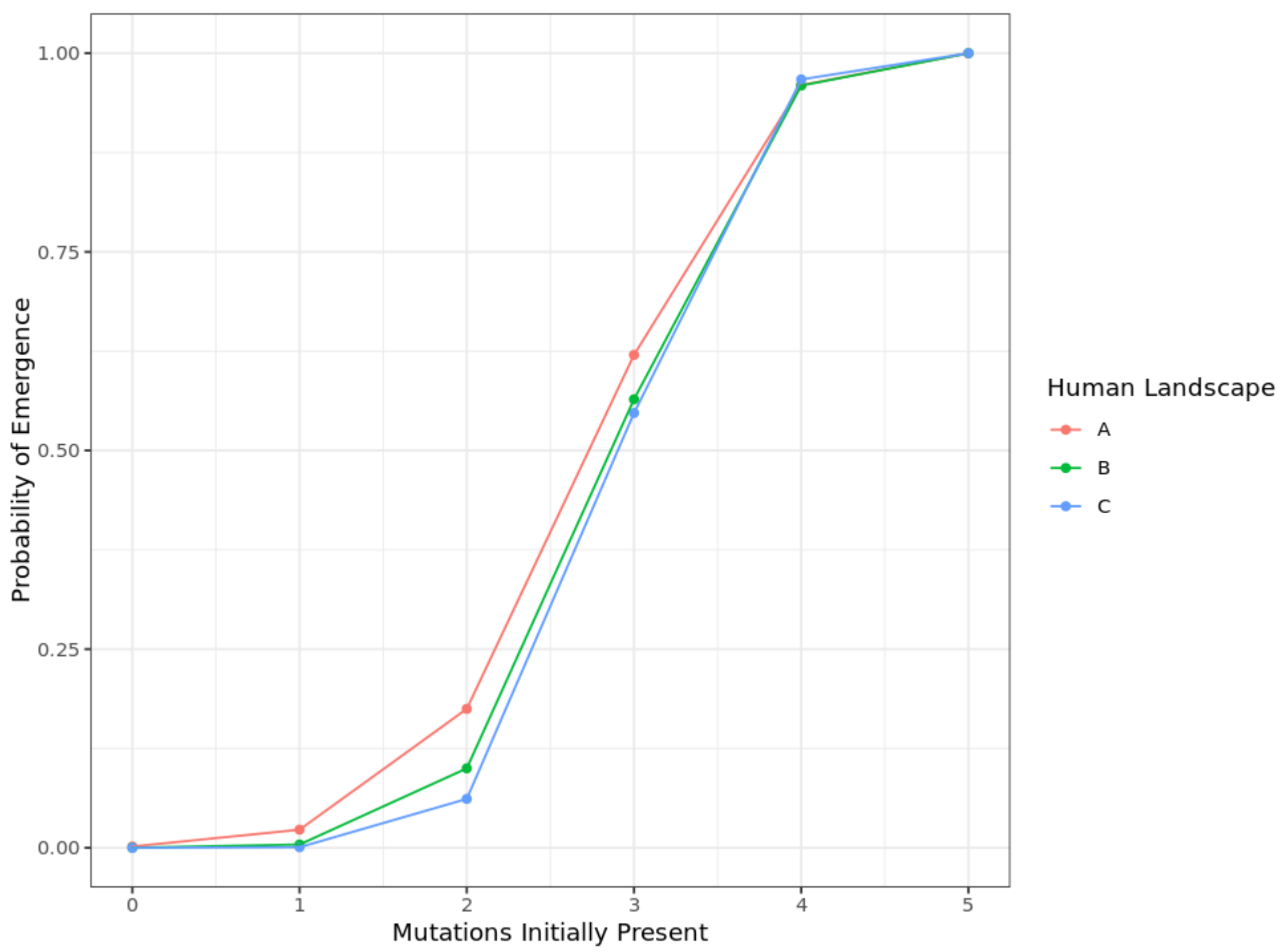

Fig. S1. Mutations and Pathogen Emergence. Probability of emergence based on number of mutations initially present for different human landscapes. Viral replication distribution is assumed to follow a normal distribution on the log 10 scale with a mean of 9 and a standard deviation of 2.

Similarly, we can look at the probability of emergence at different expected total viral population sizes under different assumptions about the zoonotic and human fitness landscape.

Algorithm S8: Comparison of emergence probabilities by number of viral replications.

```
options(repr.plot.width = 14, repr.plot.height=4)
emerge_df %>% mutate(landscapes=paste(zoo_landscape,human_landscape, sep="/")
) %>%
    ggplot(aes(x=pop_sz_log10, y=p_emerge, color=landscapes)) +
        facet_wrap(~zoo_landscape) +
        geom_line() +
        geom_point() +
        ylab("Probability of Emergence") +
        xlab("Expected Replications") +
        theme_bw()
```

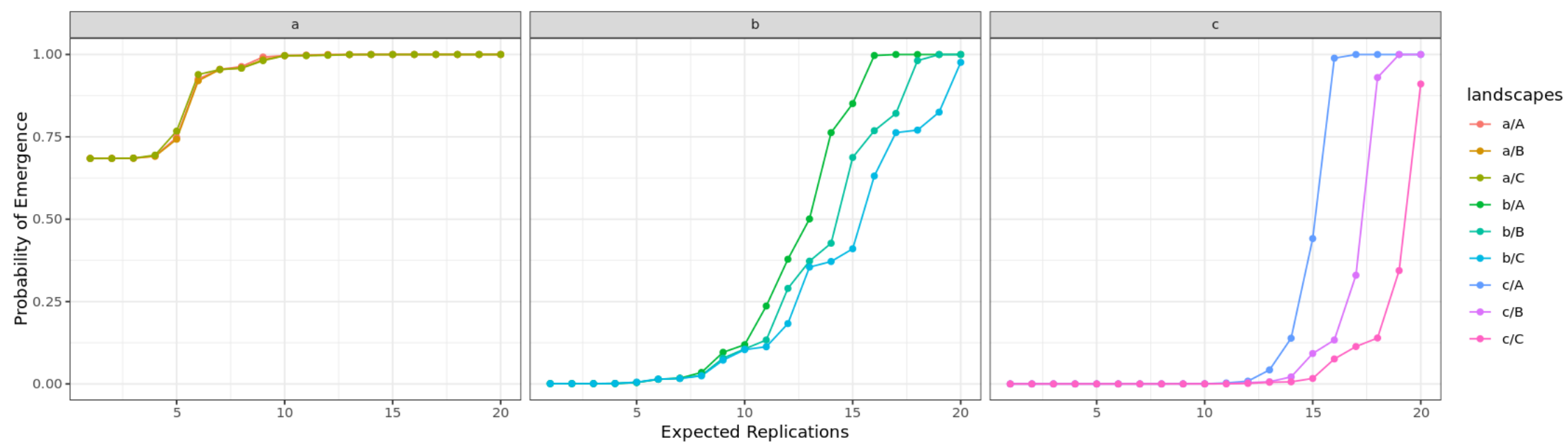

Fig. S2. Effect of replication within the human host. Probability of emergence versus number of expected replications in humans for different combinations of zoonotic and human fitness landscapes

Finally, of interest is the cross tabulation between these phenomena, namely, what is the probability of emergence after a particular number of replications in humans given the number of mutations present at introduction and the human fitness landscape.

Algorithm S9: Comparison of emergence probabilities by combination of needed mutations and number of viral replications.

```
options(repr.plot.width = 12, repr.plot.height=5)
emerge_df_initmuts %>% group_by(human_landscape, init_muts, pop_sz_log10) %>%
    summarize(p_emerge=mean(p_emerge))%>%
    ungroup()%>%
    ggplot(aes(y=pop_sz_log10, x=init_muts, fill=p_emerge)) +
        geom_tile() +
        facet_wrap(~human_landscape)+
        scale_y_continuous(breaks = 1:20) +
        scale_x_continuous(breaks=0:5) + ylab("log viral replications") +
        xlab("initial mutations present") +
        labs(fill = "F(emerge)") +
        guides(fill = guide_colorbar(title.position = "top", title.hjust = 0.
5)) +
         theme_minimal() +
        theme(legend.position="bottom")
```

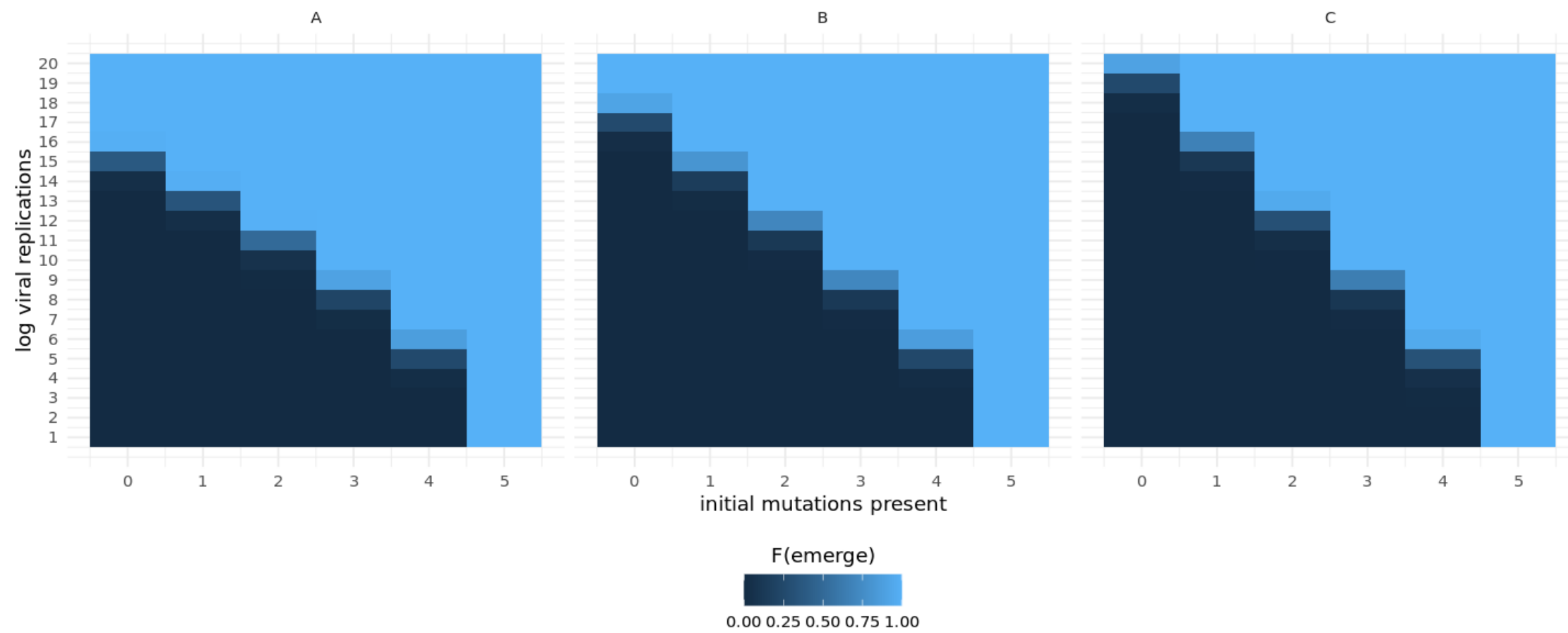

Fig. S3. Characteristics of emergence Emergence probability by number of viral replications and initial mutations initiall present for different human fitness landscapes

As a simple summarization, let's look at the same plot but only for human pattern B.

Algorithm S10: Comparison of emergence probabilities by combination of needed mutations and number of viral replications focused on human pattern B.

```
options(repr.plot.width = 5, repr.plot.height=6)


emerge_df_initmuts %>%
    filter(human_landscape=="B") %>%
    group_by(init_muts, pop_sz_log10) %>%
    summarize(p_emerge=mean(p_emerge))%>%
    ungroup()%>%
    ggplot(aes(y=pop_sz_log10, x=init_muts, fill=p_emerge)) +
        geom_tile() +
        scale_y_continuous(breaks = 1:20) +
        scale_x_continuous(breaks=0:5) + ylab("log viral replications") +
        xlab("initial mutations present") +
        labs(fill = "F(emerge)") +
        guides(fill = guide_colorbar(title.position = "top", title.hjust = 0.
5)) +
         theme_minimal() +
        theme(legend.position="bottom") +
        geom_rect(color="red", fill=NA, xmin=-.5, xmax=5.5, ymin=6.9,ymax=11.
1, linetype=2, linewidth=1)
```

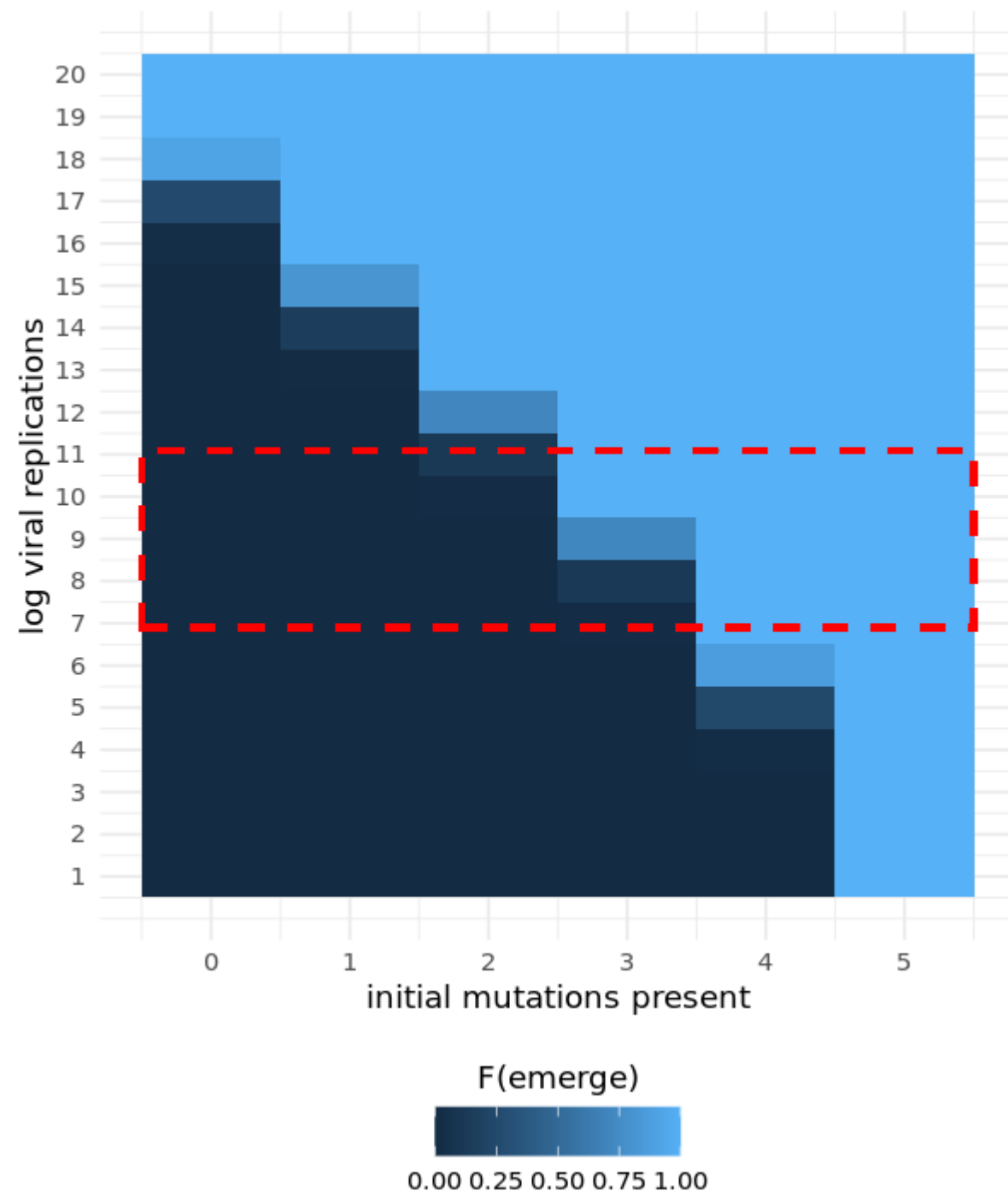

Fig. S4. Characteristics of emergence: Summary of the above used in figure 2 of the main text.

Log version

Algorithm S11: Comparison of emergence probabilities by combination of needed mutations and number of viral replications focused on human pattern B.

```
options(repr.plot.width = 5, repr.plot.height=6)

emerge_df_initmuts %>%
    filter(human_landscape=="B") %>%
    group_by(init_muts, pop_sz_log10) %>%
    summarize(p_emerge=mean(p_emerge))%>%
    ungroup()%>%
    mutate(p_emerge=as.numeric(as.character(cut(p_emerge, breaks=c(-.1,10^(-7
:0)),labels=c((-7:0))))))%>%
    ggplot(aes(y=pop_sz_log10, x=init_muts, fill=p_emerge)) +
        geom_tile() +
        scale_y_continuous(breaks = 1:20) +
        scale_x_continuous(breaks=0:5) + ylab("log viral replications") +
        xlab("initial mutations present") +
        labs(fill = "log10 F(emerge) less than") +
        guides(fill = guide_colorbar(title.position = "top", title.hjust = 0.
5)) +
         theme_minimal() +
        theme(legend.position="bottom") +
        geom_rect(color="red", fill=NA, xmin=-.5, xmax=5.5, ymin=6.9,ymax=11.
```

```
1, linetype=2, linewidth=1)
```

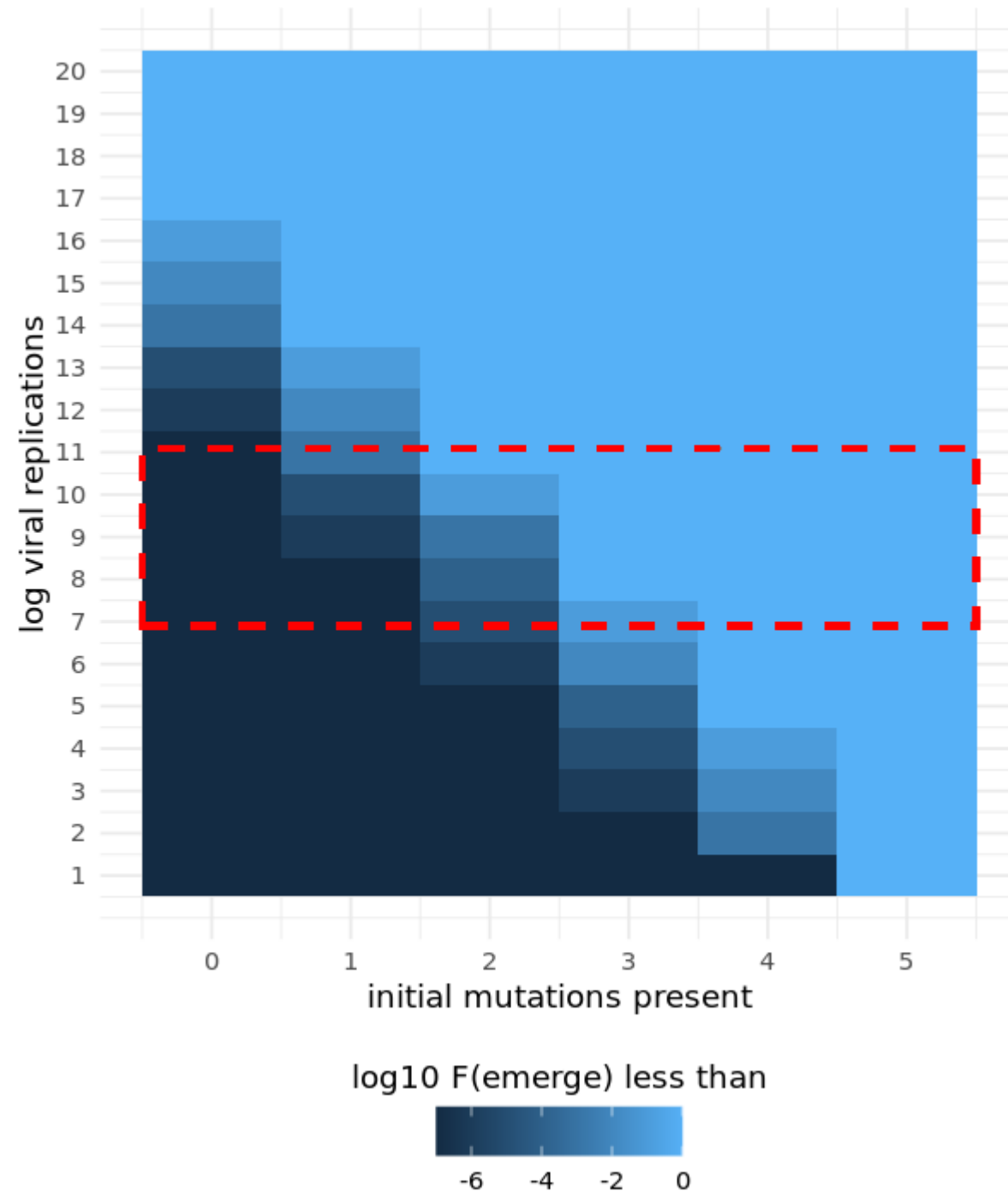

Fig. S5. Characteristics of emergence: Summary of the above on the log scale.

3. Bayesian updating based on failed emergences

Withing a Bayesian framework, we take the probabilities calculated above to help us understand how observed viral introductions into humans that fail to lead to emergence should change our beliefs about the likely evolutionary conditions and underlying prevalence of various mutation patterns in the zoonotic species.

Posterior probability of fitness landscapes

We want to calculate the probability of different fitness landscape combinations give a number failed emergences. Using the probabilities calculated above for emergence under various fitness patterns and our assumptions about the distribution of viral population sizes in humans, we can use Bayesian inference to understand what fitness landscapes are most likely.

We treat each pair of fitness landscapes (e.g., human fitness landscape A, zoonotic fitness landcape b) as a hypothesis, with equal probability of being true prior to the observation a failed emergence. Using standard Bayesian approaches, in the `rstan` package, we obtain a posterior distribution of probabilities based on the chances of seeing x failed emergence attempts under each.

Specifically, we assume the observed number of failures X follows a binomial distribution:

$$X \sim BINOM(1 - \rho_i, X)$$

where ρ_i is the probability of successful emergence under fitness landscape hypothesis i (e.g., zoonotic landscape a and human landscape B). Emergence probablities, ρ_i, are calculated through simulation as detailed in the analysis above and under the assumption the number of viral replications in a human host follows a normal distribution on a log 10 scale with a mean of 9 and a standard deviation of 2. Functionally, we assume we have a set of beliefs about the probability of each hypothesis being true, p_i, with a prior distribution of:

$$\mathbf{p} \sim Dirichlet(\boldsymbol{\alpha})$$

where $\alpha_i = 1/9$. We then use standard MCMC techniques to estimate the posterior distribution of p_i with the simplified overall observation probability (given a belief) of:

$$X \sim BINOM\left(1 - \sum_i \rho_i \, p_i, X\right)$$

It should be noted that while, here, we use a relatively simple set of canonical hypotheses, this same approach could be used for any arbitrary number of hypotheses with arbitrary complexity.

Algorithm S12: Bayesian estimation of the posterior probability of different evolutionary patterns in zoonotic and human hosts after different numbers of emergence events.

```
##Now let's run a stan model to look at the probability of different combinat
ions of fitness landscapes after a different number of failed emergences
library(rstan)
x <- 10^(0:5) #number of observed failed emergences

##Run inference on each value of x
post_dist <- list()
N <- nrow(emerge_probs)
for(i in 1:length(x)) {
    stan_data <- list(x=x[i],
        N=N,
        alpha = rep(1/N, N), ##even prior across hypotheses
        emerge_prob = emerge_probs$p_emerge
        )

    suppressWarnings(res <- stan("../code/LandscapeObsModel.stan", data=stan_
data, refresh=0)) #comment for verbose
    # res <- stan("../code/LandscapeObsModel.stan", data=stan_data) ##uncomme
nt for verbose
    chains <- rstan::extract(res)
    post_dist[[i]] <- emerge_probs %>%
        mutate(n_fails = x[i],
```

```
                post_pr_mn = colMeans(chains$p),
                post_pr_med = apply(chains$p,2,quantile, probs=0.5),
                post_pr_iqr_low = apply(chains$p,2,quantile,probs=0.25),
                post_pr_iqr_high = apply(chains$p,2,quantile,probs=0.75),
                post_pr_ci_low = apply(chains$p,2,quantile,probs=0.025),
                post_pr_ci_high = apply(chains$p,2,quantile,probs=0.975))
}

post_dist <- bind_rows(post_dist)

library(viridis)
library(scales)
library(scico)
options(repr.plot.width = 14, repr.plot.height=4)
options(scipen = 999)

post_dist %>% filter(n_fails<100000) %>%
     ggplot(aes(x=human_landscape, y=zoo_landscape, fill=post_pr_mn)) +
    geom_tile() +
    facet_wrap(~n_fails, nrow=1) +
    scale_fill_scico(transform="asn",limits=c(0,1), palette="vik",
                    direction=1, breaks=c(0,.1,.5,.9,1),
                    name="posterior\nprobability") +
    guides(fill = guide_colorbar(
        title.position = "top",
        title.hjust = 0.5,
        barwidth = 1,
        barheight = 15
    )) +
    #scale_fill_scico(transform="asn",limits=c(0,1), palette="roma", directio
n=-1) +
    geom_text(aes(label = round(post_pr_mn,5)), color = "white", size = 3.5,
fontface="bold") +
    theme_minimal() +
    ylab("Zoonotic Landscape") +
    xlab("Human Landscape")
```

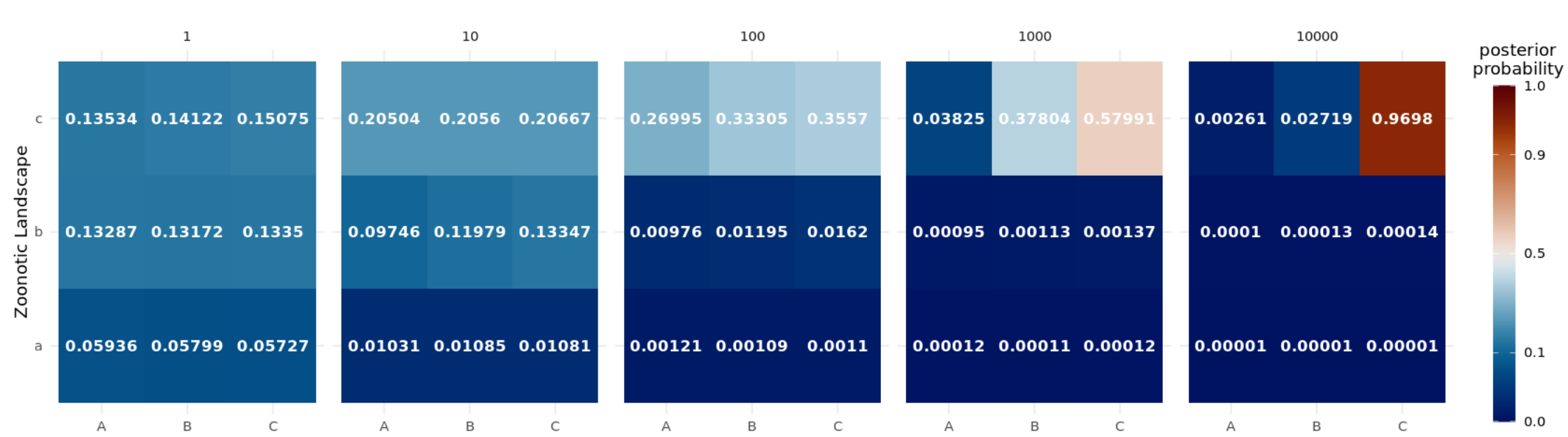

Fig. S6. Bayesian updating from spillovers. The posterior probability of different hypotheses about underlying fitness landscapes under after observing 1, 10, 100, 1,000 and 10,000 huan infections not resulting in emergence.

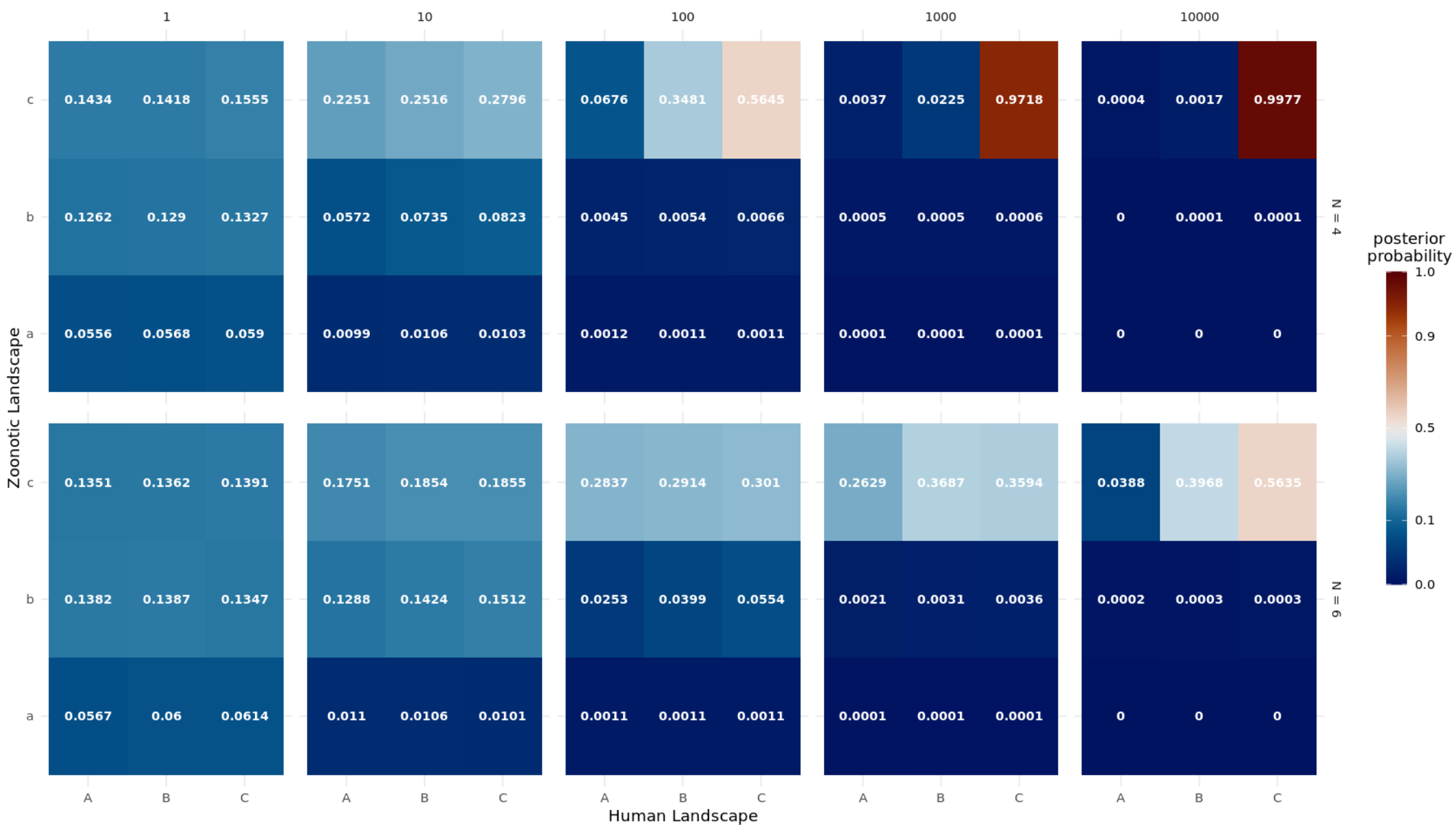

Fig. S7. Bayesian updating from spillovers with different required mutations. Posterior probability of fitness-landscape hypotheses for four (top row) and six (bottom row) necessary mutations, at 1, 10, 100, 1,000 and 10,000 failed introductions.

Posterior probability of zoonotic prevalence

We can use the same approach to, under assumptions about the human landscape of emergence (here assuming pattern B) to update our beliefs about the patterns of zoonotic prevalence under a prior assumption that prevalence follows the patterns suggested by neutral mutation (pattern b). We use the same basic approach as above, except that ρ_i now represents the probability of emergence in human fitness regime B given we start with i necessary mutations present, and p_i now represents the assumed prevalence of virions with i mutations in the zoonotic host population.

Algorithm S13: Bayesian estimation of the posterior probability of different prevalences of mutations in zoonotic hosts after observed failed emergences.

```
local_ep <- filter(emerge_probs_initmuts, human_landscape=="B")


##Run inference on each value of x
post_dist_prev <- list()
N <- nrow(emerge_probs)
```

```
for(i in 1:length(x)) {
    stan_data <- list(x=x[i],
        N=6,
        alpha = zoo_b_prevs_critmuts$p , ##nutral evolution
        emerge_prob = local_ep$p_emerge
        )
    suppressWarnings(res <- stan("../code/SimpleObsModel.stan", data=stan_dat
a, refresh=0)) ##comment for verbose
    #res <- stan("../code/SimpleObsModel.stan", data=stan_data, refresh=0) ##
uncomment for verbose
    chains <- rstan::extract(res)
    post_dist_prev[[i]] <- local_ep %>%
        mutate(n_fails = x[i],
               post_pr_mn = colMeans(chains$p),
               post_pr_med = apply(chains$p,2,quantile, probs=0.5),
               post_pr_iqr_low = apply(chains$p,2,quantile,probs=0.25),
               post_pr_iqr_high = apply(chains$p,2,quantile,probs=0.75),
               post_pr_ci_low = apply(chains$p,2,quantile,probs=0.025),
               post_pr_ci_high = apply(chains$p,2,quantile,probs=0.975))
}

post_dist_prev <- bind_rows(post_dist_prev)
```

```
#post_dist_prev
options(repr.plot.width = 14, repr.plot.height=8)
post_dist_prev %>%
    ggplot(aes(x=init_muts, y=post_pr_med)) +
         geom_bar(stat="identity", fill="lightblue")+
     geom_point() +
     geom_linerange(aes(ymin=post_pr_iqr_low, ymax=post_pr_iqr_high),
alpha=.33)+
     facet_wrap(~n_fails)
```

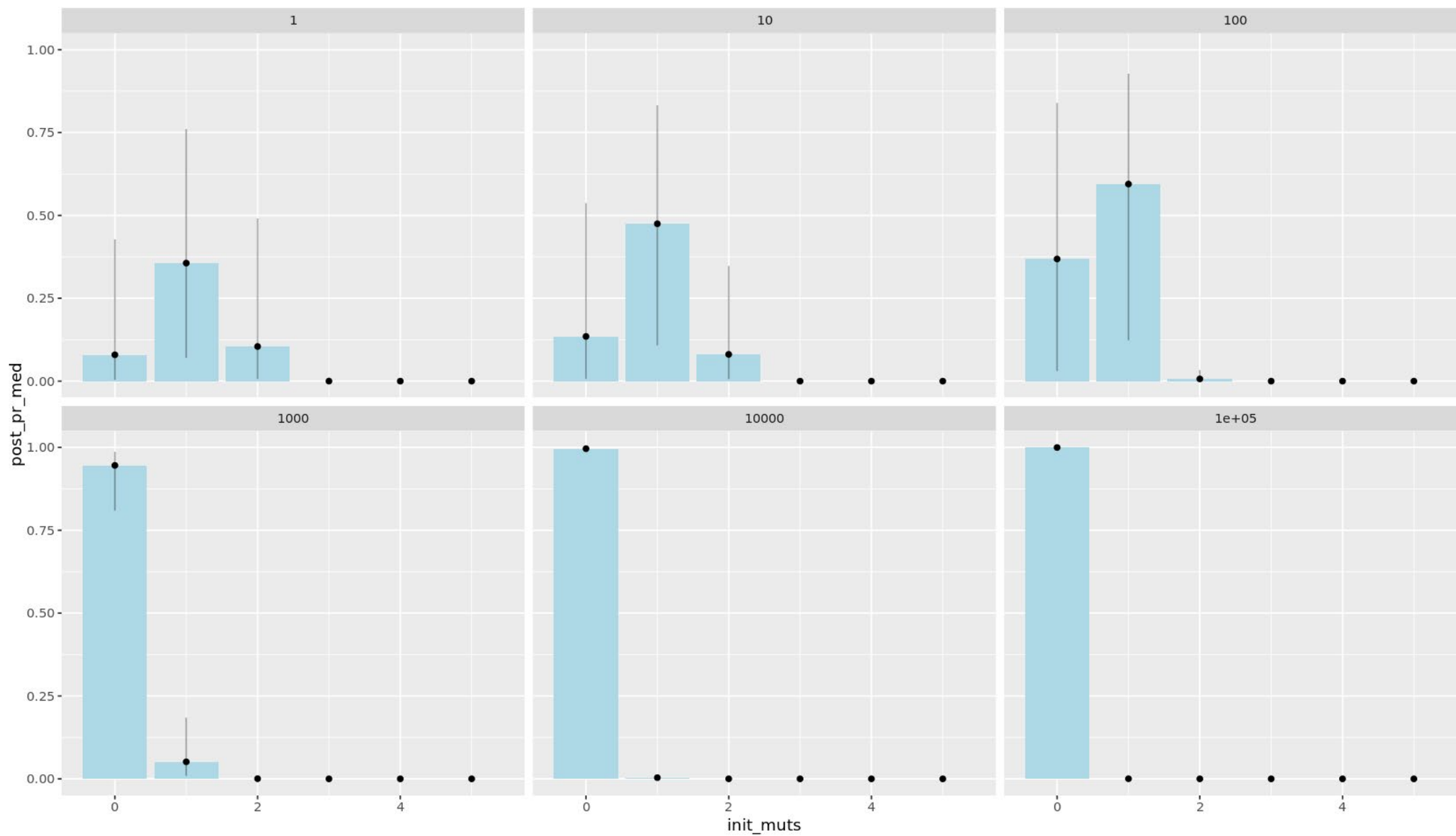

Fig. S8. Characterizing circulation in the reservoir Posterior distribution of proportion of zoonotic viral population with certain numbers of critical mutations afer 1, 10, 100, 1,000, 10,000 and 100,000 failed emergences

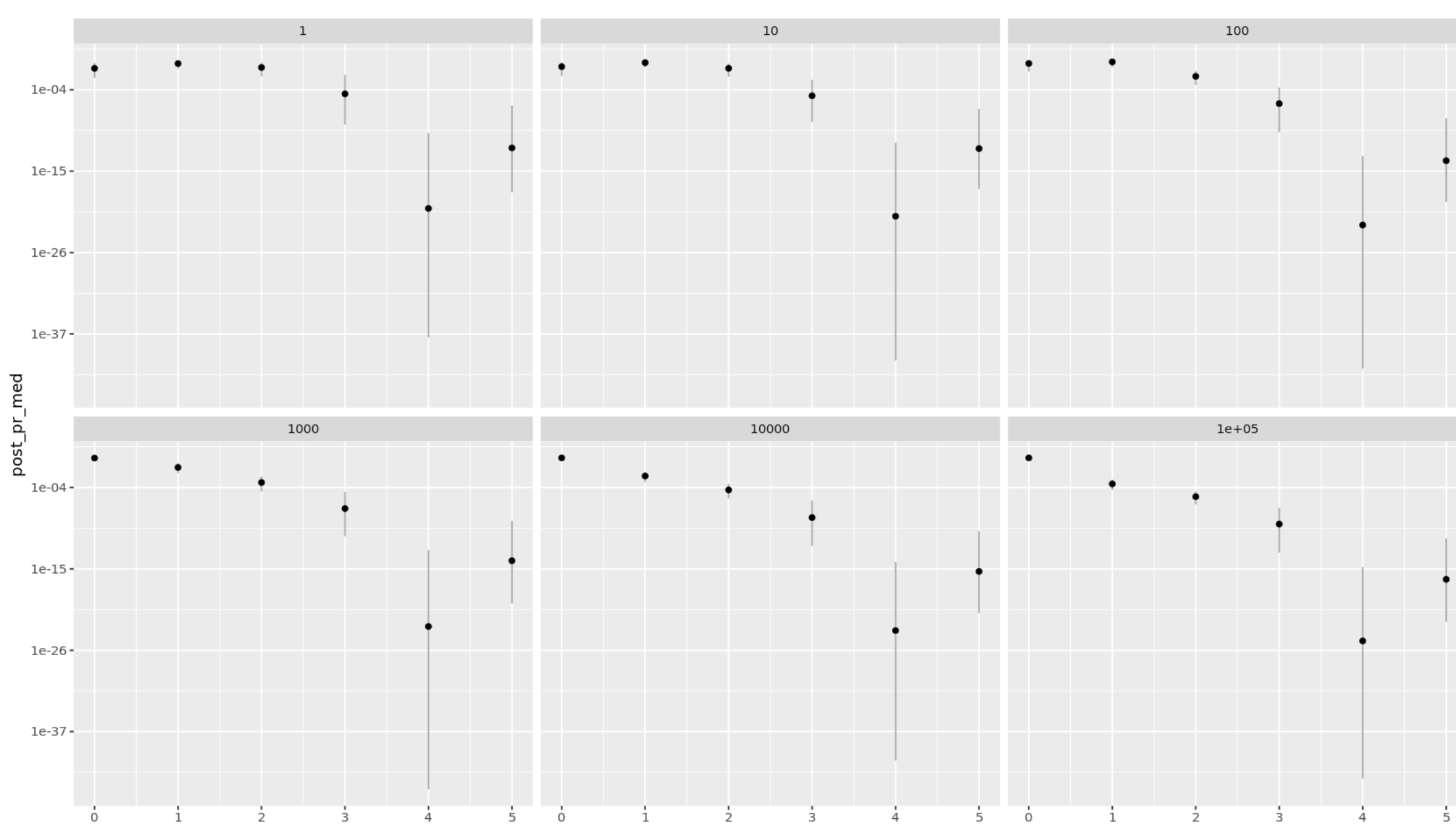

Fig. S9. Characterizing circulation in the reservoir. Posterior distribution (log scale) of proportion of zoonotic viral population with certain numbers of critical mutations after 1, 10, 100, 1,000, 10,000 and 100,000 failed emergences
